# Detecting contaminated birthdates using generalized additive models

**DOI:** 10.1186/1471-2105-15-185

**Published:** 2014-06-12

**Authors:** Wei Luo, Marcus Gallagher, Bill Loveday, Susan Ballantyne, Jason P Connor, Janet Wiles

**Affiliations:** 1 Centre for Pattern Recognition and Data Analytics, Deakin University, Geelong, Australia; 2 School of Information Technology and Electrical Engineering, The University of Queensland, Brisbane, Australia; 3 Drugs of Dependence Unit, Queensland Health, Brisbane, Australia; 4 Discipline of Psychiatry, The University of Queensland, Brisbane, Australia; 5 Centre for Youth Substance Abuse Research, The University of Queensland, Brisbane, Australia

## Abstract

**Background:**

Erroneous patient birthdates are common in health databases. Detection of these errors usually involves manual verification, which can be resource intensive and impractical. By identifying a frequent manifestation of birthdate errors, this paper presents a principled and statistically driven procedure to identify erroneous patient birthdates.

**Results:**

Generalized additive models (GAM) enabled explicit incorporation of known demographic trends and birth patterns. With false positive rates controlled, the method identified birthdate contamination with high accuracy. In the health data set used, of the 58 actual incorrect birthdates manually identified by the domain expert, the GAM-based method identified 51, with 8 false positives (resulting in a positive predictive value of 86.0% (51/59) and a false negative rate of 12.0% (7/58)). These results outperformed linear time-series models.

**Conclusions:**

The GAM-based method is an effective approach to identify systemic birthdate errors, a common data quality issue in both clinical and administrative databases, with high accuracy.

## Background

Birthdate information is ubiquitous in clinical, administrative, and research databases. It is one of the most common pieces of information for identifying individuals. Unfortunately, also common in these databases are missing or incorrect birthdates. For example, we have previously identified 1.5% of birthdates as incorrect in a state-wide public health service data set with more than 20,000 patient records [1]. Similarly, in a study of 1112 hospital admissions 2% of patients were registered with incorrect names or birthdates [2]. These errors are typically more common in paediatric databases [3].

In most databases, birthdates are stored as date-time values. Compared to a numeric data field, there are a number of reasons date-time columns may be more prone to contamination during data collection and transfer. Firstly, a date-time can be communicated in more than one geographical standard format: The date “January 2nd, 2010” can be entered into a database as “2010-01-02” (ISO8601 standard), “01/02/2010” (North American format), “02/01/2010” (British/Australian format), or even “02-Jan-10” (default format in Oracle 9i). If a registration clerk uses a format inconsistent with the database, incorrect birthdates will be generated. These format inconsistencies frequently result in truncated dates or NULL values in the database. Secondly, suboptimal user-interface design can induce incorrect birthdates. For example, some user interfaces remember a date previously entered and use it to auto-complete the input textbox. If a registration clerk does not check carefully, an incorrect value may be repeatedly entered. As data are often entered from paper record, direct validation is often difficult. Finally, patients themselves may give an incorrect birthdate, considering the emergency nature of many patient encounters. For example, a patient may confuse age with the year of birth (e.g. age 53 being turned into 1953 as the year of birth) or round the birthdate to the first date of the birth year (e.g., “June 25, 1910” being turned into “January 1, 1910”). Parents or caregivers of young or elderly patients admitted may also be prone to birthdates errors.

As birthdate information is central in patient identification, incorrect birthdates may have severe consequences, for example transfusion of blood or marrow to the wrong patient [4]. Incorrect birthdates may also cause records to be overwritten or duplicated, which may eventually incur significant financial loss to the hospital [3]. Finally incorrect birthdate information can affect derived variables. If an incorrect birthdate occurs repeatedly, then age-based risk stratification based on the data would be invalid [5].

Similar to other data quality issues, the best solution for reducing incorrect birthdates is to have good quality assurance at the data collection stage [6]. However, as health care data is often collected in busy clinical environments, data quality can be less that optimal. It is therefore critical to identify and correct the incorrect birthdates in existing data. Given the volume and complexity of patients seen in both the public and private health systems, it is no longer possible to manually check complete patient databases. With the rapid growth of electronic health records, tools are needed to automatically identify likely erroneous birthdates.

Despite the prevalence of incorrect birthdates and the potential cost and adverse health outcomes that can result, few tools are available to identify birthdate errors. Often incorrect birthdates are identified in an ad-hoc fashion, using rules specific to a given database and its use. For example, one common type of birthdate errors is that the NULL representing a missing birthdate is replaced by a date representing zero. The detection of such an error often relies on prior knowledge of the zero encoding [7].

If incorrect birthdates were generated in a completely random fashion, it would be impossible to identify them. However in most databases, the majority of the incorrect birthdates are introduced by a few sources of common contamination. In the previous zero-encoding example, the missing birthdates are often replaced by a zero date when data is transferred from one owner to another. The replaced value depends on the software involved and therefore is not random. For example, Microsoft products often use “December 30, 1899” as the zero date-time, and Unix (ISO 8601) uses “January 1st, 1970 UTC” as the zero date-time.

More generally, when birthdates in a database are contaminated systematically, we expect the database to acquire artefacts in the birthdate distribution, in particular over-representation of certain birthdates. Based on this observation, we propose an effective approach to identify systemic contamination of birthdates.

The importance of planned data cleaning has long been recognized in the research community. When the database size is not an issue, interactive data cleaning is always a good starting point [8]. From the perspective of data warehousing, Rahm and Do proposed a taxonomy of data cleaning problems [9]. They first identified two levels of data cleaning problems: Schema level and Instance level. Then for each level, they defined different scopes and problems. Birthdate contamination fits into the “Misspelling” problem within the “Attribute” scope at the Instance level. In a wider context, Van den Broeck and colleagues defined data cleaning as a 3-stage process: Screening/Detecting, Diagnosis, and Editing [5]. This paper deals with the Detecting stage of data cleaning. In philosophy, our proposed method fits into the general strategy of checking for inconsistencies reflected in frequency distributions and strange patterns. However, because the prevalence of birthdate contamination, its special characteristics, and the threats it poses to data quality, separate attention is needed for detecting birthdate contamination, in particular in a live large database environment.

## Methods

### Distribution of birthdates in a database

For the purpose of this paper, we consider only databases that identify one or more groups of individuals—such as patients or clinicians. Many clinical or administrative databases fall into this category including electronic health records (EHR), emergency department information systems, databases of controlled drug prescriptions, and medical claims databases. With a clear identification of individuals in such a database, each date *d* defines a set of *N*(*d*) individuals born on that day.

In an ideal situation where the actual numbers of births and deaths for every day are available, for example in a region with a well-maintained birth and death registry and limited population migration, the actual number of births in the population for every date can be estimated. But with any given database, it is useful to distinguish 1) the general population *P*_1_ that includes everyone living in a region at a certain time period, 2) the “at risk” population *P*_2_ that includes everyone who in theory could be included in the database (e.g., males to a prostate cancer database), and 3) the group of people *P*_3_ who are actually in the database. Individuals in a database *P*_3_ can be regarded as a sample of the “at risk” population *P*_2_. Consequently the number *N*(*d*) can be regarded as realization of a birthdate distribution defined on *P*_2_.

In an ideal situation where the actual numbers of births and deaths for every day are available, for example in a region with a well-maintained birth and death registry and limited population migration, the actual number of births in the population for every date can be estimated. But with any given database, it is useful to distinguish Consider an event that an individual was born at time *t* and later included in *P*_3_. We assume that such an event follows a Poisson point process with a time-varying intensity function *μ*(*t*). In a Poisson point process with intensity function *μ*(*t*), for any time interval *A*, the number of events *N*_*A*_ in *A*, follows a Poisson distribution $p\left(N_{A}=k\right)=\frac{\lambda {\left(A\right)}^{k}e^{-\lambda \left(A\right)}}{k!}$, where $\lambda \left(A\right)=\int_{t\in A}\mu \left(t\right)\mathrm{dt}$. Hence for a date *d*, the number of individuals with the birthdate *d* follows a Poisson distribution defined by

(1) $$p\left(N\left(d\right)=k\right)=\frac{\lambda {\left(d\right)}^{k}e^{-\lambda \left(d\right)}}{k!}.$$

Here *k* is the number of individuals born on day *d* and *λ*(*d*) = ∫_*t* ∈ *d*_*μ*(*t*)d*t* is the aggregated intensity for date *d* and equals the expected value of *N*(*d*).

In certain situations with larger data variance, a negative binomial distribution can be used instead. That requires one more over-dispersion parameter to fit. In this application, it is difficult to assess over-dispersion; hence we prefer the simpler Poisson model assumption.

Let *μ*_*d*_ be the mean of *μ*(*t*) on day *d* and *l* be the length of a day. Then *λ*(*d*) = *μ*_*d*_ ⋅ *l*. If *μ*(*t*) changes slowly, sequence < *λ*(*d*) > can be regarded approximately as the result of sampling *μ*(*t*) daily and then multiplying the sample with the constant *l*. The sequence < *λ*(*d*) > is determined by the birthdate distribution of the general population *P*_1_ and the representation of *P*_1_ in the database.

The birthdate distribution of the general population *P*_1_ is determined on a large scale by the age profile of the population and on a small scale by seasonal and weekly fluctuations in child births. The age profile of the population (as in [10]) is the aggregated result of changes in birth and death rates. For example, the post-WWII baby boom has contributed to the aging population structure in US, Canada, and Australia [11,12]. As such changes are often driven by long lasting demographic forces such as economic development, war, and progress of medical science [13,14], they manifest as slow and smooth variation in the sequence < *λ*(*d*)>. In contrast, seasonal and weekly variations in child births (as in [15]) act on a shorter temporal scale. More recently, most child births occur in a hospital environment and scheduled Caesarean section or labour induction are more frequent. These factors lead to more births on weekdays than weekends, which produces cyclic dips in the sequence < *λ*(*d*) > [16,17].

How the general population is represented in a patient database is mostly determined by the nature of the disease. For example, a database of prostate cancer patients should contain only males; a database of skin cancer patients probably contains more Caucasians than patients of other races. In terms of the birthdate distribution, one common consideration is that a disease may pose higher risk to certain age groups. For example, individuals born before year 1930 may be disproportionately represented in a database of chronic non-cancer pain. Like the population age profile, the nature of the disease acts on a larger temporal scale and it should not affect the smoothness of the sequence < *λ*(*d*)>. For example, a person born on March 2nd, 1980 and a person born on March 3rd, 1980 should have very similar chances of being included in a database, assuming all other conditions are equal.

Finally for most hospitals, a patient database grows out of paper records. As a hospital serves more patients, its database may cover a larger portion of the at-risk population *P*_2_. However, it is reasonable to assume that any such change in the size of the database will affect different age groups proportionally, and is independent of the shape of < *λ*(*d*) >.

To summarize, the birthdate distribution of a database can be modelled using a function *λ*(*d*) that is generally smooth but contains weekly dips due to weekend reduction in births.

### Tell-tale indicators of birthdate contamination: discrepancy between the expected and observed counts

When birthdates in a database are systematically contaminated, for example when a missing value is consistently replaced with the zero date-time, incorrect birthdates may be repeatedly generated. In such cases, the frequency of an incorrect birthdate *d* will be higher than other dates in the database. That is, the *observed* number of patients with birthdate *d*, denoted *N*(*d*), will be larger than the *expected* number *λ*(*d*). Therefore identifying incorrect birthdates can be achieved through identifying the date *d* whose person count *N*(*d*) is well above the expected count *λ*(*d*). Of course this observation is not new. For example, the difference between *N*(*d*) and *λ*(*d*) is called *within deviation* by Dasu and Johnson [18]. To the authors’ knowledge, however, no previous attempt has been made to statistically model this discrepancy between *N*(*d*) and *λ*(*d*) for error detection.

The deviation of *N*(*d*) from the expected count *λ*(*d*) can be measured in terms of the tail probability of the Poisson distribution. This provides a way to rank outliers across all birthdates. The top *N*(*d*)s with the smallest tail probabilities can be returned to the data cleaning staff for further confirmation and investigation. Alternatively, a small positive number *c* can be used as a threshold: *N*(*d*) is then labelled as an outlier if

$$\sum_{k=N\left(d\right)}^{\infty }p\left(k|\lambda \left(d\right)\right)=\sum_{k=N\left(d\right)}^{\infty }\frac{\lambda {\left(d\right)}^{k}e^{-\lambda \left(d\right)}}{k!}<c.$$

In many applications, only a small number of the most likely erroneous birthdates will be manually checked. If required, the expected number of birthdate errors in database can be estimated from the results of manually checking small samples of the database [19].

### A generalized additive model for birthdate distribution

The expected birthdate distribution *λ*(*d*) can be estimated from the data < *N*(*d*) > by assuming smoothness of the function *λ*(*d*), with proper handling of the reduction in births on weekends. In the previous section, we have seen that explicit modelling of the mechanisms shaping the age profile would be very complex. Nevertheless, both long-term and seasonal variations in births/deaths can be recovered by smoothing of counts *N*(*d*) in a relatively short temporal window. The reduction in weekend births can be modelled by a multiplicative factor that only affects Saturdays, Sundays, and public holidays. As the reduction is a gradual development driven by increasing hospital births, elective Caesarean section, and labour induction, the multiplicative factor can also be modelled by a smooth function. Finally, it is worth noting that government policies to encourage fertility can also lead to more births on a particular day [20], but such events are very rare and should be explicitly modelled on a case-by-case basis.

Different techniques can be used to smooth the raw data < *N*(*d*) > to recover the function *λ*(*d*). But to explicitly model the day-of-week effect, we use an additive model in which *λ*(*d*) is modelled as the product of an overall smooth function and a multiplicative factor for weekends. (A justification for the weekend multiplicative factor is that the weekend births are moved from the weekend to the preceding week). We assume that

(2) $$\text{log}\left(\lambda \left(d\right)\right)=s_{1}\left(d\right)+I\left(d\;\text{is in weekend}\right)\cdot s_{2}\left(d\right).$$

Here the log link function is used because *λ*(*d*) can only be positive; *I*(⋅) is the indicator function and *s*_1_(*d*) and *s*_2_(*d*) are smooth functions of *d*. The function *s*_1_(*d*) models both long-term and seasonal variations in the birthdate distribution; *s*_2_(*d*) models the gradual change in the reduced weekend births. Public holidays are similar to weekends; it only requires extra bookkeeping in the modelling process.

Following the standard practice in generalized additive models, the smoothing terms in Equation (2) are represented by regression splines [21]. That is, $\;\hat{s}\left(x\right)=\sum_{i=1}^{q}b_{i}\left(x\right)\beta_{i}$, where *b*_*i*_(*x*) are a set of basis functions. Common type of basis functions include B-splines and more familiar cubic splines. These basis functions are sections of polynomials that join at a number of knot locations. To avoid manually selecting knots for the regression splines, thin-plate splines can be used [22]. With *n* data points, a thin-plate spline representation is as follows.

$$\hat{s}\left(x\right)=\sum_{i=1}^{n}\delta_{i}\eta_{\mathrm{md}}\left(\left|\left|x-x_{i}\right|\right|\right)+\sum_{j=1}^{M}\alpha_{j}\phi_{j}\left(x\right)$$

Here *d* is the dimension of the function domain (in our case *d* = 1), *m* is the order the smoothness penalty, *ϕ*_*j*_ are $M=\left(\begin{array}{c} m+d+1\\ d \end{array}\right)$ basis functions that spans the null space of a penalty energy function, and *η*_*md*_ is a radial-basis function. ***δ*** = (*δ*_1_, *δ*_2_, …, *δ*_*n*_)^*T*^ and ***α*** = (*α*_1_, *α*_2_, …, *α*_*M*_)^*T*^ are parameters with the constraint $\sum_{i=1}^{n}\delta_{i}\phi_{j}\left(x_{i}\right)=0$ for each *j*. Further details of thin-plate splines can be found in [23]. Just like other kernel methods, the computation of thin-plate splines has a complexity of *O*(*n*^3^). For ease of computation, a lower rank approximation of ***δ*** is used. Such reduced-rank thin-plate splines [23] are used in our application. The rank of thin-plate splines controls the smoothness of the function, and is selected by generalized cross-validation criterion (GCV) [24]. To capture seasonal variations in birthdate distribution, the maximum rank for the candidate splines should be set to a sufficiently large number. We recommend a maximum rank of 2 *m*, where *m* is the number of years covered by the birthdates.

**Figure 1:**
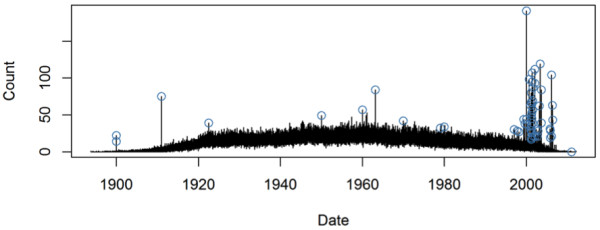
**The number of individuals in a database grouped by birthdate, shown as a time series.** Some large numbers are known to be caused by incorrectly replacing missing birthdates with some fill-in values. These are annotated with blue circles.

Dasu and Johnson [18] generated a histogram similar to Figure 1 to help detect inadvertent censoring caused by a default date. Graphical features of the histogram such as spikes and V-shaped valleys were identified as indicators for missed or censored data. However, the presence and location of such graphical features are determined by visual inspection. In contrast, here with a GAM, the deviation of particular counts in the histogram can be quantified for probability based decisions.

## Results

### A public health application

We use a health administration database to demonstrate the fitting of GAM and the identification of outliers. A drug regulatory authority maintains a database of drug dispensing records, which covers around 40*,*000 individuals with birthdates ranging from the 1890s to 2010s. The data is integrated from separate data collections from more than 3000 off-site pharmacies. As each pharmacy may choose different software to manage their data, quality assurance at the data collection stage is limited. For example, one system replaces the null date-time value with a default date, which changes whenever the system is updated. This has resulted in a typical downstream database with multiple sources of contamination. Our task was to identify those birthdates that may have been incorrectly introduced by the faulty data management software. As the data were transcribed from doctor’s prescriptions filled at off-site (community) pharmacies, they were geographically and functionally independent of the data custodians. These handwritten paper scripts were archived after data entry. Geographically locating, accessing and then physically combing through these handwritten documents is theoretically possible (with generous resources), but was impractical for the purposes of this study.

Figure 1 shows the number *N*(*d*) of individuals for each birthdate *d*. For evaluation purposes, most incorrect birthdates have already been manually identified by a domain expert. There were 58 errors in total. The authors have no knowledge of the criteria used by the domain expert to identify the errors and how complete and accurate they are. Nevertheless, Figure 1 shows at least three potential sources of database errors. First on the far left, errors 1899-12-30 and 1900-01-01 were likely to have been introduced by incorrect handling of the zero date-time in software systems. The error 1911-01-01 may also have resulted from the confusion of year 1911 and year 2011. Second in the middle, errors 1950-01-01, 1960-01-01, 1963-02-14, 1970-01-01, 1979-01-01, 1980-01-01 may result from incorrect self-reported birthdates. Finally on the right of the figure, a group of incorrect birth dates were introduced by the pharmacy management system that mixed birthdates with drug dispensing dates.

### Outliers detected through generalized additive model

**Figure 2:**
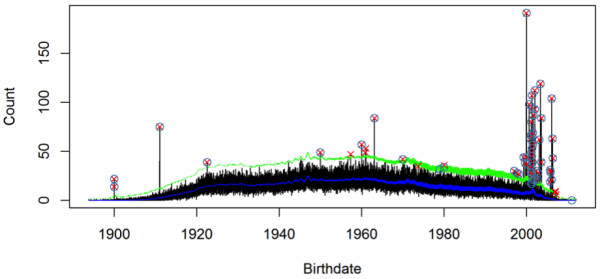
**Outliers returned by the non-homogeneous Poisson model.** The blue line shows the estimated Poisson intensity function. The green dashed line shows the point-wise 99.99th percentile of the Poisson distribution. The estimated outliers are in red crosses and the outliers identified by the domain expert are in blue circles. In total 59 outliers are identified, resulting in 8 false positives and 7 false negatives.

We fit an additive model in the form of Equation (2) to the birthdate counts in Figure 1. The gam function from the mgcv R-package [23] was used to estimate the intensity function *λ*(*d*). The smoothness was determined through GCV with an upper bound of 200 for the degree of freedom. Figure 2 shows the estimated intensity function and the outliers above the point-wise 99*.*99th percentile.

The detection resulted in 8 false positives and 7 false negatives. The false positives can be classified into two categories: 5 in the middle between years 1957 to 1980; 3 on the far right between years 2006 and 2007. From visual inspection, the 5 false positives in the middle are indeed outliers. We conjecture that these birthdates were caused by multiple identities of some patients. When data from multiple pharmacies were linked, patient identities were consolidated through names and addresses. Mismatch can happen during the consolidation process and the patient’s birthdate would be overrepresented. The 3 false positives on the right are also curious. They seem to suggest that there are dozens of very young (~6 years old) patients on controlled drugs. This warrants further investigation.

The 7 false negatives include 2 in the middle (1979-01-01 and 1980-01-01), 4 on the right (all in year 2001), and 1 on the right (2011-01-01). The false negative 2011-01-01 has too few patients to justify a statistical decision. The 4 false negatives in year 2001 coincide with a large number of incorrect birthdates that have been correctly identified. We believe that after the correctly identified errors have been cleaned, these 4 false negatives will likely be found by rerunning the identification procedure.

Because the sequence < *N*(*d*) > forms a time series, nonparametric smoothing [25] as a common way to find outliers in time series can also be used. However, Figure 2 shows three advantages of our model-based approach over nonparametric smoothing:

1) Explicit background knowledge such as the weekend effect is simply modelled. The reduced weekend birth is reflected in the increased variation of the green line in the right part of Figure 2. If the smoothing was done with standard nonparametric methods, such background knowledge is not easy to be incorporated—it would be difficult to select a smoothing parameter (bandwidth) that works well with the changing variation in the signal. Nonparametric models are not good at handling the non-normality of count data.

2) Percentile estimation (the green line in Figure 2) can be easily extracted from the parametric Poisson model. Most nonparametric smoothing methods assume Gaussian distribution of residuals, which is not appropriate for percentile estimation of count data.

3) Compared with direct smoothing, the model-based approach is robust at the two ends of the birthdate range, where the data are sparse and *N*(*d*)s for most days equal zero. In nonparametric smoothing, consecutive zeros at the two ends will result in both zero signal estimate and zero variance estimate, which renders every nonzero *N*(*d*) an outlier. For example, in nonparametric smoothing based on the mean absolute deviation (MAD), the median of all neighbouring points of *d* is used to estimate the underlying signal and the median of all absolute deviation measures the variance [25]. For a sequence (0, 0, 0, 900, 0, 1, 0), the median is 0 and the median of all absolute deviation is also 0. Hence both 900 and 1 will be estimated to be outliers. In contrast, with a Poisson distribution, the probability *P*(*k* ≥ 1|*λ*(*d*)) can still be large with even a low intensity *λ*. This will greatly minimize the likelihood of false positives at the two ends of the age spectrum.

**Figure 3:**
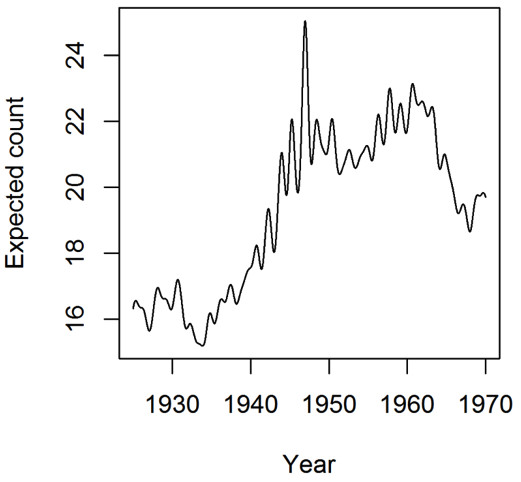
**Estimated poisson intensity λ(d) for weekdays between 1925 and 1970.** It reveals a seasonal pattern of child birth. It also shows a period of decreased birth rate during the ‘Great Depression’ (in the 1930s) and a period of increased birth rate during the post-war baby boom (between 1946 and 1961).

The estimated Poisson intensity function *λ*(*d*) (the blue line in Figure 2) reflects both the general age profile of the patient population and short-term variations in demographics. Figure 3 shows the segment of the curve for weekdays between year 1925 and 1970. The curve suggests a seasonal pattern of child birth. The dip around 1933–1934 suggests the effect of the ‘Great Depression’ on child birth in Australia. The elevated counts at the centre echo the baby boom in Australia following the second world war, which Salt defined to be the period between 1946 and 1961 [12]. The latter two features of the estimated function *λ*(*d*) are consistent with the official statistics [26].

**Figure 4:**
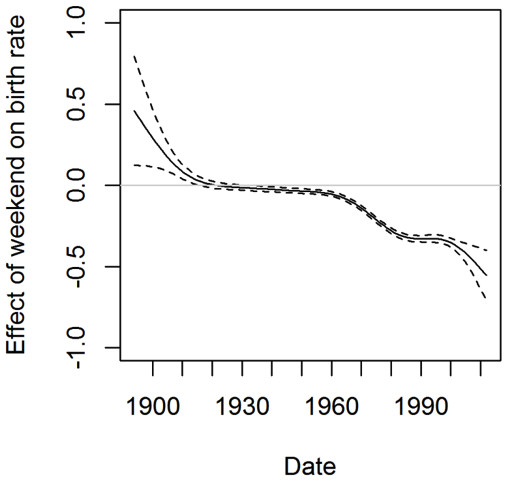
**The smoothing term *s***_**2**_**(*d*) in Equation (2).** The dashed lines show 95% confidence interval of the estimate. It suggests that before year 1920, there were more weekend births than weekday births. The number of weekend births has decreased since year 1960, very likely due to wider adoption of elective Caesarean section.

The effect of reduced weekend birth (term *s*_2_(*d*) in Equation (2)) is shown in Figure 4. It is consistent with the trend that weekend births have been significantly reduced due to elective Caesarean section. It also suggests that elective Caesarean section has been gaining popularity in the past 50 years.

### Comparison with outlier detection based on a standard time-series model

As the sequence < *N*(*d*) > is also a time series, an autoregressive integrated moving average (ARIMA) model is a natural alternative to a generalized additive model. An **ARIMA(p, n, q)** model contains *p* autoregressive terms, *n* nonseasonal differences, and *q* moving average terms. It has the following mathematical expression: $\left(1-\sum_{i=1}^{p}\phi_{i}L^{i}\right){\left(1-L\right)}^{n}N\left(d\right)=\left(1+\sum_{i=1}^{q}\theta_{i}L^{i}\right)\varepsilon_{d}$, where *L* is the lag operator, *ϕ*_*i*_ and *θ*^*i*^ are autoregressive and moving average parameters, respectively. When a time series is season with *m* points per season, a seasonal ARIMA (SARIMA) model can be used. In a **SARIMA(p, n, q)(P, N, Q)**_**m**_ model, additional P autoregressive terms, N differences, and Q moving average terms are used to model the seasonality of the time series. More details of ARIMA models can be found in most time series text books (e.g., [27]).

Although ARIMA models may be more familiar to many people, they are not ideal for modelling the birthdate distribution. First, an ARIMA model assumes normality of data, which is not always appropriate as *N*(*d*) is a count. In particular, outlier detection based on normal percentiles may not be as accurate compared with Poisson percentiles. Next, it is not easy to incorporate other covariates in an ARIMA model and assess their effects on the counts. Most importantly, from a linear time series model like ARIMA, it is impossible to infer point-wise tail probabilities (see the green line in Figure 2). If an over-all tail probability estimate is used, incorrect birthdates at the two ends of the age range will be missed (see the third advantage of the GAM described previously).

**Figure 5:**
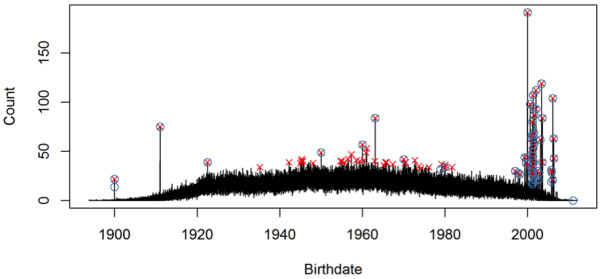
**Outliers returned by the seasonal ARIMA model.** Assuming normality of the residuals, birthdates with residuals above the 99.99th percentile are identified as outliers. The estimated outliers are in red crosses. In total 69 outliers are identified, resulting in 22 false positives and 11 false negatives.

We fit a seasonal ARIMA (SARIMA) model on the sequence < *N*(*d*) > adjusting for the weekly cycle. It results in a high-order model **ARIMA(0, 1, 5)x(0, 0, 2)**_**7**_. By looking above the 99.99^th^ percentile in the residuals, we identified 69 outlier birthdates (See Figure 5). We compared these birthdates with the birthdates identified by the domain experts. A total of 36 birthdate errors were correctly matched, with 11 false negatives and 22 false positives, mostly in the period between year 1940 and 1980, in which many patients were born. Because no point-wise percentile can be inferred, this method tends to miss the birthdate errors near the left end of the birthdate range, where the data is sparse. For example, the date 1900-01-01 was identified by the GAM model (see Figure 2), but was missed by the ARIMA model. A comparison of sensitivity and specificity of the two methods are shown in Table 1.

**Table 1:** Sensitivity and specificity of the two outlier detection methods based on GAM and SARIMA

**Outlier detection**	**Sensitivity**	**Specificity**
GAM-based	0.8793	0.9998
SARIMA-based	0.5345	0.9994

The GAM-based method showed better performance in terms of sensitivity and specificity. The area under the curve (AUC) for SARIMA-based outlier detection was 0.991; AUC for GAM-based outlier detection was 0.998 (p-value 0.28).

### Age profile of young oxycodone user

Finally, we show how identification of incorrect birthdates can prevent serious misinterpretation of data.

Oxycodone is an opioid analgesic used for pain management. A patient using oxycodone for an extended period is likely to develop dependence to the medication. Therefore in many places, including the state of Queensland in Australia, an oxycodone treatment episode longer than two months requires a report to the regulatory authority. Here we use the drug dispensing database to understand the age distribution of long-term users of oxycodone.

**Figure 6:**
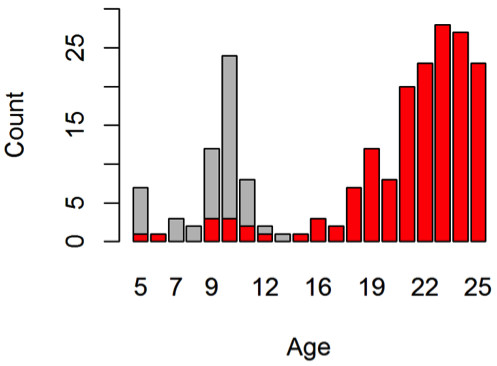
**Age distribution of patients whose annual consumption of oxycodone exceeds 4500 mg.** It shows unusual clusters at age 5 and 10. The red bars highlight the estimated age distribution after records with suspicious birthdates removed.

The age distribution for younger patients is shown in Figure 6^a^. The figure shows unusual clusters of patients of age 5 or 10. These clusters are worrying as the main reason for Oxycodone prescription is chronic non-cancer pain, which is rare among children.

We applied the GAM-based identification method to find birthdate errors. The 99*.*99th percentile was used as the cut-off. The records with identified birthdate errors were then removed from the analysis. The corrected age distribution based on the cleaned subset is highlighted in red. Although the new distribution still shows several young patients whose presence in the database is worth further investigation, it is more consistent with the common understanding that young people rarely have chronic pain condition that warrants large quantities of opioids.

## Discussion

In current data-quality practice, data artefacts are typically identified by simple cross-tabulation or visual inspection [6]. However, visual inspection is only feasible if the database contains only a limited number of distinct values. Visual inspection also relies on arbitrary cut-offs and it is difficult to bound the false negative rate. The GAM model effectively addressed these two problems.

As the importance of data quality becomes more widely recognized, the proposed method provides a new tool for data quality improvement. The method can be implemented to run automatically in most commercial databases and the results can be easily incorporated into regular reports on data. For many downstream databases, data quality reports allows one to identify quality problems at the earliest time possible and contain contamination of the upstream data.

Our method could be further improved with external data, in particular from well-curated sources. Population wide birth and death statistics are often available from government organisations. For example, the Australian Bureau of Statistics provides the numbers of births by year and month. Potentially such data can augment a model solely based on the counts in a database. A way to use the population wide statistics is to devise a Bayesian prior distribution based on the external data.

In extreme situations, an incorrect birthdate may induce a very large peak in the birthdate sequence, similar to the examples in the top right of Figure 1. These outliers may bias the estimation of the intensity curve itself, reducing the model’s power to detect other less extreme outliers. This is often known as the *masking effect* in the outlier detection literature [28]. In cases of extreme outliers, one option is to refit the GAM model with the identified outliers removed. Alternatively, a robust GAM fitting procedure [29] can be used. As robust GAM fitting requires intense computation, further work is needed so that it can be applied in a large database with a wide age range.

In view of the limitations discussed above, we see two lines of future research that will generate immediate benefits to a broad range of applications. First, when an external data source is available, it would benefit from concrete techniques for using the external data in birthdate distribution modeling. External data may provide two types of anxillary information: the general shapes of the age distribution and the degree of variance from one day to another. A technique to incorporate such information would generate better models. Second, when a robust smoothing procedure is required, it would benefit from efficient algorithmic implementation. As multicore and cluster systems are becoming more common, parallel algorithms that tailor towards such computing facilities may be desirable.

## Conclusions

Birthdates are the most commonly collected domain of health information. Their accuracy is critical to effective health service delivery. Our results demonstrate that a GAM model achieves efficacy and flexibility in detecting incorrect birthdates caused by systematic contamination. The GAM model described in this paper provides a solution that works across a diverse range of health databases.

## Endnote

^a^To tell whether a patient use oxycodone for an extended period of time, a simple way is to calculate the total quantity of the medication consumed in a year. World Health Organization (WHO) has guidelines of the defined daily dose (DDD [24]) for an individual: The DDD for oxycodone is 75 mg per day. Hence if the annual consumption of a patient exceeds the DDD equivalent quantity of two months (4500 mg), then we assume that the patient is a long-term oxycodone user.

## Competing interests

The authors declare that they have no competing interests.

## Authors’ contributions

WL and JW conceived of the study. WL carried out the experiment and analyzed the results. All authors participated in its design and coordination and helped to draft the manuscript. All authors read and approved the final manuscript.
